# Implantable cardiac monitors: the digital future of risk prediction?

**DOI:** 10.1093/ehjdh/ztae036

**Published:** 2024-06-11

**Authors:** Axel Bauer, Clemens Dlaska

**Affiliations:** Department of Cardiology, Medical University of Innsbruck, Anichstr. 35, 6020 Innsbruck, Austria; Department of Cardiology, Medical University of Innsbruck, Anichstr. 35, 6020 Innsbruck, Austria

**This editorial refers to ‘Dynamic risk stratification of worsening heart failure using a deep learning-enabled implanted ambulatory single-lead electrocardiogram’, by J. Howard *et al*., https://doi.org/10.1093/ehjdh/ztae035.**

An implantable cardiac monitor (ICM) can certainly be regarded as a small marvel of technology. Weighing less than 3 g, it can monitor a person’s rhythm for years. In clinical practice, ICMs are currently used for long-term rhythm monitoring after clinical events such as syncope, stroke, or ablation therapy, but this small device can perhaps do much more. The single-lead ambulatory electrocardiogram (aECG) recorded by the device represents a valuable bio-signal whose information may offer far more than a mere analysis of the heart rhythm.

In recent years, groundbreaking work has impressively demonstrated the unexpected potential of ordinary ECGs that can be unleashed by the power of artificial intelligence (AI). A particularly interesting development in this context is that AI methods enable the usage of the ECG as a deep phenotyping tool.^1^ For example, AI-based ECG algorithms can detect traces of past atrial fibrillation episodes in a sinus rhythm ECG,^2^ identify incipient or existing left ventricular dysfunction,^3^ or diagnose aortic valve stenosis.^4^ However, most algorithms were based on the analysis of high-quality 12-lead ECGs.

In the current issue of the journal, Howard and colleagues report on a possible substantial expansion of how ICMs might be used in the future. The authors focused on a significant clinical problem: Early detection of worsening of heart failure (HF) leading to hospitalization. The authors first developed an AI algorithm (aECG-CNN) based on a deep convolutional neural network (CNN) that allowed for detection of poor left ventricular function [left ventricular ejection fraction (LVEF) ≤ 40%] from aECGs. To achieve this goal, the authors elegantly linked Medtronic’s data warehouse to electronic health records data sets of US hospitals, which resulted in a database of 49 073 aECG-LVEF pairs from 3749 individual patients. Splitting the data randomly at the patient level for training (35 741 aECG-LVEF pairs from 2249 patients), validation (6721 aECG-LVEF pairs from 750 patients), and testing (6611 aECG-LVEF pairs from 750 patients), the algorithm yielded a good performance with an area under the receiver operator characteristic curve of 0.80 on the test data set. However, recognizing that poor LVEF is not synonymous with HF worsening (HF patients with poor LVEF can be clinically stable), the authors postulated that greater variability in aECG-CNN outputs may signal impending HF hospitalization. This hypothesis was tested in a real-world cohort of 909 patients with implanted ICMs and previous HF diagnosis. For every patient and every month, time series features from the daily aECG-CNN values were computed to capture aECG-CNN variability. Of the 12 467 monthly evaluations, 201 preceded a HF hospitalization event in the subsequent month. The findings revealed that months categorized as high-risk based on aECG-CNN variability (15% of the months) were 1.9 times more likely to experience HF hospitalization compared with those deemed low risk.

The authors should be commended for conducting this interesting study, which is innovative in several respects: One, establishing a robust database, both in qualitative and quantitative terms, is not only a fundamental necessity but also a significant challenge for any deep learning-driven strategy. In this study, the authors successfully integrated two extensive data sources, each managed by different custodians, necessitating a thorough Health Insurance Portability and Accountability Act-compliant procedure. Two, the use of AI methods to interpret ICM signals for the prediction of clinical conditions, such as reduced LVEF, is an innovative approach that opens new clinical perspectives. However, while the algorithm underwent sufficient validation across distinct data sets, it awaits further external validation with entirely independent data sets for reinforced credibility and evaluation at the level of single patients (rather than at the episode level). In addition, the presented HF risk stratification approach should be compared with an entirely AI-based method, i.e. an AI model that is trained to directly predict the risk of future HF, as for example has been developed in the context of atrial fibrillation.^5^ Finally, the present study showcases the effectiveness of dynamic risk assessment methodologies for rapidly changing conditions like HF. Such an approach has also been proven effective in the randomized SMART-MI trial^6^ in patients after myocardial infarction in which ICM-based detection of asymptomatic yet significant arrhythmic events (including atrial fibrillation, higher-degree atrioventricular-block, and non-sustained ventricular tachycardia) allowed for an effective real-time reclassification of risk.

It should be noted that the patient population of the present study may not directly reflect HF patients encountered in routine clinical settings. The LVEF was surprisingly high at 53%, as was the proportion of patients with atrial fibrillation (57%), diabetes mellitus (39%), and high blood pressure (95%). At the same time, only a minority of patients received up-to-date medical treatment that would be expected in a HF population (e.g. 55% were administered angiotensin converting enzyme inhibitors, angiotensin receptor blockers, or angiotensin receptor/neprilysin inhibitors, and a mere 25% received spironolactone). Thus, the patient population likely represents a HF with preserved ejection fraction cohort. The applicability of these findings to a HF with reduced ejection fraction cohort remains uncertain. Additionally, the fact that patients received an ICM for clinical reasons (24% for atrial fibrillation, 18% post-stroke, 38% following syncope, and others) may introduce a selection bias. Those limitations could be overcome by a prospective study, which would be necessary before this technology should be used clinically.

**Figure 1:**
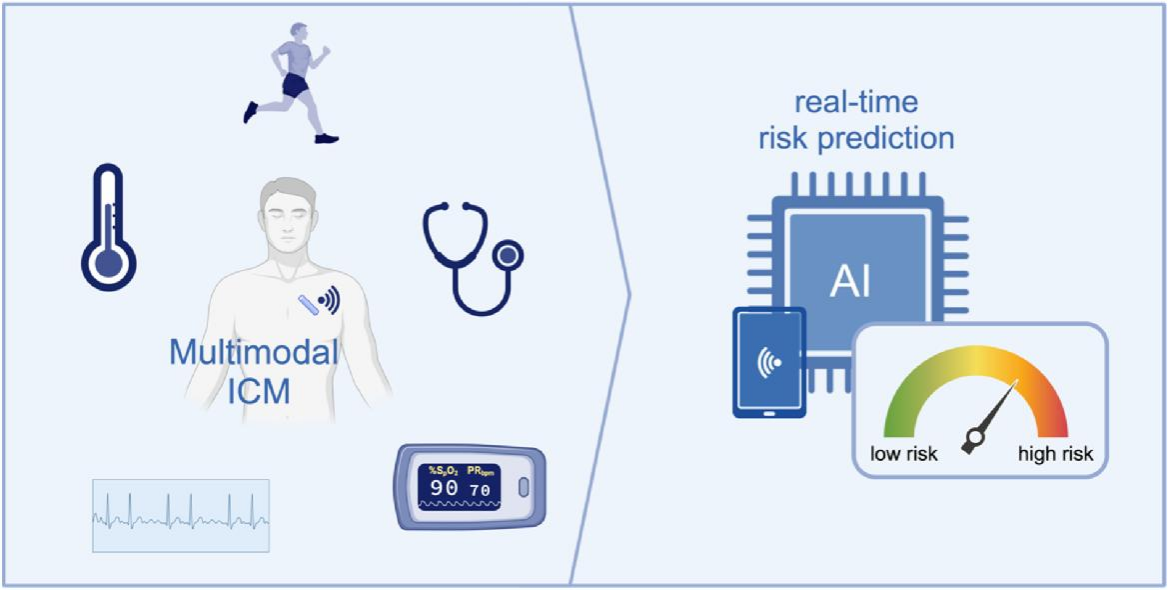
Multimodal signal acquisition by next generation implantable cardiac monitors will enable real-time artificial intelligence-driven risk assessment. AI, artificial intelligence; ICM, implantable cardiac monitor. Created with biorender.com.

In summary, the research conducted by Howard and colleagues compellingly indicates the high potential of digital ICM-based risk prediction. Future generations of ICMs could be further developed to record a wider range of physiological data in addition to the heart’s electrical signals, such as respiration, physical activity, body temperature, heart sounds, and oxygen saturation (*Figure 1*), which would allow for a multimodal real-time risk assessment. But even today, the combination of the continuous monitoring capabilities of ICMs with the powerful analytical potential of AI represents a successful synergy.
